# Cough hypersensitivity in patients with metabolic syndrome: a clinical finding and its possible mechanisms

**DOI:** 10.1186/s12890-021-01652-x

**Published:** 2021-09-06

**Authors:** Jiafen Cheng, Zhuangli Xie, Shengyuan Wang, Siwan Wen, Shanshan Niu, Cuiqin Shi, Li Yu, Xianghuai Xu

**Affiliations:** 1 Department of Nephrology, Shanghai Tenth People’s Hospital, School of Medicine, Tongji University, No. 301 Yanchangzhong Road, Shanghai, 200072 China; 2 Center for Nephrology and Clinical Metabolomics, Shanghai Tenth People’s Hospital, School of Medicine, Tongji University, No. 301 Yanchangzhong Road, Shanghai, 200072 China; 3 Department of Endocrinology and Metabolism, Tongji Hospital, School of Medicine, Tongji University, No. 389 Xincun Road, Shanghai, 200065 China; 4 Department of Pulmonary and Critical Care Medicine, Tongji Hospital, School of Medicine, Tongji University, No. 389 Xincun Road, Shanghai, 200065 China

**Keywords:** Metabolic syndrome, Cough sensitivity, Obstructive sleep apnea–hypopnea syndrome, Gastroesophageal reflux disease, Airway inflammation

## Abstract

**Purpose:**

To investigate the changes of cough sensitivity in patients with metabolic syndrome and its possible mechanisms.

**Method:**

A total of 29 metabolic syndrome (MetS) patients with OSAHS (group-1), 22 MetS patients without OSAHS (group-2), and 25 healthy controls (group-3) were included. All participants underwent a routine physical examination and completed the gastroesophageal reflux disease questionnaire (GerdQ), and the inflammatory mediator profile were determined. The cough threshold for capsaicin, induced sputum cell count and cell classification, and inflammatory mediators in induced sputum supernatants were compared. The correlation between capsaicin cough sensitivity and various indicators in the MetS population was analyzed.

**Results:**

The minimum concentration of inhaled capsaicin needed to induce ≥ 5 coughs (C5) was significantly different among three groups (H = *14.393, P* = 0.001) and lower for group-1 and group-2 than it for group-3 (*P* = 0.002, *P* = 0.005). The percentage of neutrophils in induced sputum and the concentrations of calcitonin gene-related peptide (CGRP), substance P (SP), and interleukin 8 (IL-8) in the sputum supernatant of group-1 and group-2 were significantly higher than those of group-3. Besides, the pepsin concentrations were significantly different among the 3 groups (F = 129.362, *P* < 0.001), which significantly was highest in group-1 (*P* < 0.001) and lowest in group-3 (*P* < 0.001). Triglycerides, AHI, pepsin concentration and BMI were risk factors of increased capsaicin cough sensitivity.

**Conclusion:**

Increased capsaicin cough sensitivity in MetS patients is closely related to sleep apnea and gastroesophageal reflux. For patients in MetS patients without OSAHS, gastroesophageal reflux is an important factor for increased capsaicin cough sensitivity. Airway inflammation, especially airway neurogenic inflammation, may also play a role in the pathogenesis of increased capsaicin cough sensitivity.

*Trial registration* The protocol was registered in the Chinese Clinical Trials Register (http://www.chictr.org.cn/) (ChiCTR1800014768). Written informed consent was obtained from all participants before enrollment.

## Introduction

Chronic cough is defined as cough ≥ 8 weeks with normal chest X-ray or CT scan. Cough variant asthma, upper airway cough syndrome, eosinophilic bronchitis, and gastroesophageal reflux-induced cough are common etiologies of chronic cough [1, 2]. Obstructive sleep apnea–hypopnea syndrome (OSAHS) has recently been considered as a possible disease that can lead to chronic cough [3]. Most cough could be controlled or relieved after treatments according to the diagnosis and treatment procedures [4]. However, 10–42% of chronic cough patients cannot clarify the etiology or receive effective treatment measures [5], causing refractory or persistent cough. Therefore, rare etiologies must be continuously identified, which can effectively reduce the proportion of patients with refractory cough.

Studies have found that obese asthmatic patients have a unique asthma phenotype, which is characterized by a higher level of inflammation of neutrophils in sputum and blood [6]. Morales-Estrella et al*.* reported that patients with a higher BMI experience cough more often than other patients and that the cough is more severe in obese patients [7], indicating that obese patients an increased susceptibility to cough. Recently, it was suggested that type 2 diabetes may be a risk factor for chronic cough and that self-reported chronic cough was more common in diabetic patients than in the general population [8]. Our previous research showed that OSAHS patients have a predisposition to cough hypersensitivity associated with airway inflammation [9]. Since obesity, type 2 diabetes and sleep apnea are all closely associated with chronic cough and bronchial asthma, metabolic syndrome (MetS) should be considered.

Because increased cough sensitivity is a common feature of all chronic cough patients, it is also called cough hypersensitivity syndrome [10, 11]. We hypothesized that MetS patients had high cough sensitivity and were more vulnerable to coughing after receiving endogenous or exogenous coughing stimuli. This study tested the hypothesis by comparing the capsaicin cough sensitivity, induced sputum cell counts, and inflammatory mediators in induced sputum supernatants among MetS patients with OSAHS, MetS patients without OSAHS, and healthy control populations and explored the possible mechanisms underlying the increased capsaicin cough sensitivity in MetS patients.

## Materials and methods

### Participants

1. MetS with OSAHS group: A total of 29 MetS patients diagnosed in the Department of Endocrinology and Metabolism at Tongji Hospital from December 2017 to May 2020 were included in this group. Moreover, these patients were diagnosed with OSAHS by polysomnography (PSG).

2. MetS without OSAHS group: A total of 22 MetS patients who were diagnosed in the Department of Endocrinology and Metabolism at Tongji Hospital from December 2017 to May 2020 were included in this group. In addition, these patients were included in this group, and did not meet OSAHS after being diagnosed by PSG.

Diagnostic criteria for MetS: MetS patients were investigated according to the “Chinese guidelines on the Prevention and Treatment of Dyslipidemia in Adults” revised in 2007 by the joint committee that developed the guidelines [12]: (1) abdominal obesity—waist circumference ≥ 90 cm for males and ≥ 85 cm for females; (2) hyperglycemia—fasting blood glucose ≥ 6.1 mmol/L or blood glucose ≥ 7.8 mmol/L 2 h after glucose load and/or diagnosed with diabetes and subsequently treated; (3) hypertension—blood pressure ≥ 130/85 mmHg (1 mmHg = 0.133 kPa), and/or confirmed hypertension and receiving treatment; (4) fasting triglyceride (TG) ≥ 1.70 mmol/L; and (5) fasting high-density lipoprotein cholesterol (HDL-C) < 1.04 mmol/L. Patients who meet 3 or more criteria can be diagnosed with MetS.

Diagnostic criteria for OSAHS: OSAHS was diagnosed according to the “International Classification of Sleep Disorders (3rd edition)” by the American Sleep Association (ICSD-3, 2014) [13].

3. Healthy control group: During the same period, 25 healthy volunteers without allergy were enrolled from the staff and medical students in the hospital and designated as healthy control group.

The inclusion criteria for the above participants were as follows: (1) between 18 and 65 years of age; (2) no wheezing, hemoptysis, and fever; (3) no obvious abnormalities on chest X-ray or CT; (4) pulmonary function tests: forced expiratory volume in one second (FEV_1_) > 80% of predicted value, and FEV_1_/forced vital capacity (FVC) > 70%; and normal airway reactivity; and (5) tolerable to cough sensitivity test to capsaicin and sputum induction. The exclusion criteria were as follows: (1) smoking history or smoking cessation < 2 years; (2) respiratory infections within 3 months; (3) pregnant or lactating women; (4) mental health disorders. In addition, all participants with a history of chronic cough were also excluded.

The protocol was registered in the Chinese Clinical Trials Register (http://www.chictr.org.cn/) (ChiCTR1800014768). Written informed consent was obtained from all participants before enrollment.

### Method

1. Capsaicin cough sensitivity: Capsaicin (30.5 mg) was dissolved in Tween 80 (1 mL) and ethanol (1 mL) and then dissolved in physiological saline (8 mL) to provide a stock solution of 1 × 10^−2^ M, which was stored at − 20 °C. This solution was diluted with physiological saline to make solutions of 0.49, 0.98, 1.95, 3.9, 7.8, 15.6, 31.2, 62.5, 125, 250, 500 and 1000 µM. Each subject inhaled a control solution of physiological saline, followed by progressively increasing concentrations of the capsaicin solution. Solutions were inhaled for 15 s every 60 s, by tidal mouth-breathing wearing a noseclip from a Bennett Twin nebulizer (3012–60 cc; Puritan-Bennett Co., Carlsbad, CA, USA) operated by compressed air at 5 L/min. Increasing concentrations were inhaled until five or more coughs were elicited. The nebulizer output was 0.21 mL/min. It has been reported that aerodynamic mass median diameter (MMD) of the particle is 3.60 µM, with a geometric standard deviation of 3.47 [14, 15]. Capsaicin-induced cough number was counted by two medical technicians in the pulmonary function laboratory. Cough threshold C5 was defined as the lowest concentration of capsaicin that elicited five or more coughs.

2. Epworth sleepiness scale (ESS): According to the ESS proposed by Johns [16], a total score < 5 points was defined as normal, 5–9 points was defined as mild sleepiness, 10–15 points was defined as moderate sleepiness, and 16–24 points was defined as severe sleepiness.

3. Gastroesophageal reflux disease questionnaire (GerdQ) [17]: The questionnaire comprises 6 symptom-related questions, of which 4 are questions related to symptoms positively correlated with reflux (A1, A2, C1, and C2) and the other 2 are questions related to symptoms negatively correlated with reflux (B1 and B2). The questionnaire asks respondents to recall the frequency of each symptom in the past week, and symptoms are divided into 4 levels according to degree. The score for each positive symptom increases with the increasing frequency of symptom onset. No positive symptoms appearing within the past week was set at 0 point; a positive symptom appearing 1 day within the past week was set at 1 point; a positive symptom appearing 2–3 days within the past week was set at 2 points; and a positive symptom appearing 4–7 days within the past week was set at 3 points. The scoring criteria for negative symptoms were opposite, and the frequency grades were set as 3, 2, 1, and 0 points. The GerdQ score was the sum of scores for each symptom; the score ranged from 0 to 18 points, and a total score ≥ 8 points indicated the possibility of gastroesophageal reflux disease. The higher the score was, the higher the possibility.

4. Induced sputum cell counts: The analysis was conducted according to a method established by Department of Pulmonary and Critical Care Medicine at Tongji Hospital [18]. Briefly, the subjects continuously inhaled the 4% hypertonic saline solution through an ultrasonic nebulizer (YS9801, Yisheng Corp., Shanghai, China) and were asked to expectorate sputum into a sterile pot every 5 min, after blowing their noses and rinsing their mouths. Sputum with minimal salivary contamination was immediately collected, mixed with 4 volumes of 0.1% dithiothreitol by gentle aspiration and then was mixed on a bench rocker for 20 min. The filtrate through a 48-mm gauze was centrifuged at 3000 rpm for 10 min, and the cell-free supernatant was removed and stored at − 80 °C until assay. The cell pellet was re-suspended in 1 mL of PBS and smeared on glass slides, followed by total cell counting using a standard hemocytometer. The air-dried preparations were stained with HE stain, and then cell differential was performed on 400 nucleated cells according to standard morphological criteria.

5. Analysis of inflammatory mediators in induced sputum supernatants: The levels of bradykinin, calcitonin gene-related peptide (CGRP), substance P (SP), pepsin, prostaglandin E2 (PGE2), histamine, eosinophil cationic protein (ECP), and interleukin-8 (IL-8) in induced sputum supernatants were analyzed according to the kit instructions. The average value of 3 measurements was used as the final result. Each reagent kit was provided by Shanghai Yuanxiang Medical Devices Co. Ltd., improting from R & D Systems (United States). The error within the same detection batch was < 5%, and the error among batches was < 10%. The lower limit of detection for each mediator was as follows: bradykinin, 39.0 pg/mL; CGRP, 7.8 pg/mL; SP, 2.5 ng/mL; pepsin, 0.01 ng/mL; PGE2, 0.01 pg/mL; histamine, 0.78 ng/mL; ECP, 1.5 ng/mL, and IL-8, 7.8 pg/mL.

6. Measurement of basic parameters: The basic parameters including height, weight, waist circumference and blood pressure were measured by professionally trained medical personnel. Height data were accurate to 0.1 cm and body weight was accurate to 0.1 kg. Waist circumference was measured according to the method recommended by the World Health Organization (WHO) and the data were accurate to 0.1 cm. Blood pressure measurement was performed twice at an interval of 5 min, and the average was taken.

7. Measurement of blood biochemical parameters: All subjects fasted for more than 10 h, and fasting venous blood was drawn the morning of the next day. Triglycerides, HDL-C, and blood glucose were measured in our laboratory according to routine procedures.

8. Pulmonary function tests and exhaled nitric oxide measurement: These procedures were conducted by professional technicians. Pulmonary function tests were performed using a MasterScreen spirometer (Jaeger, Germany) according to the instructions developed by the American Thoracic Society [19]. Exhaled nitric oxide measurements were performed according to the manufacturer’s manual (NIOX, Sweden). Pulmonary function and histamine bronchial provocation tests were performed according to the methods recommended by the Respiratory Branch of the Chinese Medical Association. The instruments adopted were MasterScreen Diffusion lung function instrument and APS nebulizer from Jaeger Company (Germany). With histamine as the stimulant, when the cumulative histamine dose (PD20-FEV1) that reduces FEV1 by 20% was less than 7.8 mol, the increased airway reactivity was considered. All indeterminate results of the above tests were conducted for a second time, and conclusive results were finally got. No significant adverse events occurred during above tests.

9. PSG monitoring: All night sleep monitoring was performed using Alice 5 Polysomnography (Philips Respironics, Inc.). Nose and mouth airflow, percutaneous oxygen saturation (S_p_O_2_), electroencephalography (EEG), eye movement, mandibular electromyography (EMG), chest and abdomen respiratory motion, posture, leg movements and snoring were recorded for at least 7 h. After monitoring, the sleep physician (Dr. Shi) manually analyzed and normalized the data to calculate the apnea–hypopnea index (AHI, the average number of apneas or hypopneas recorded during the study per hour of sleep), minimum oxygen saturation during sleep, and mean oxygen saturation.

### Process

**Fig. 1:**
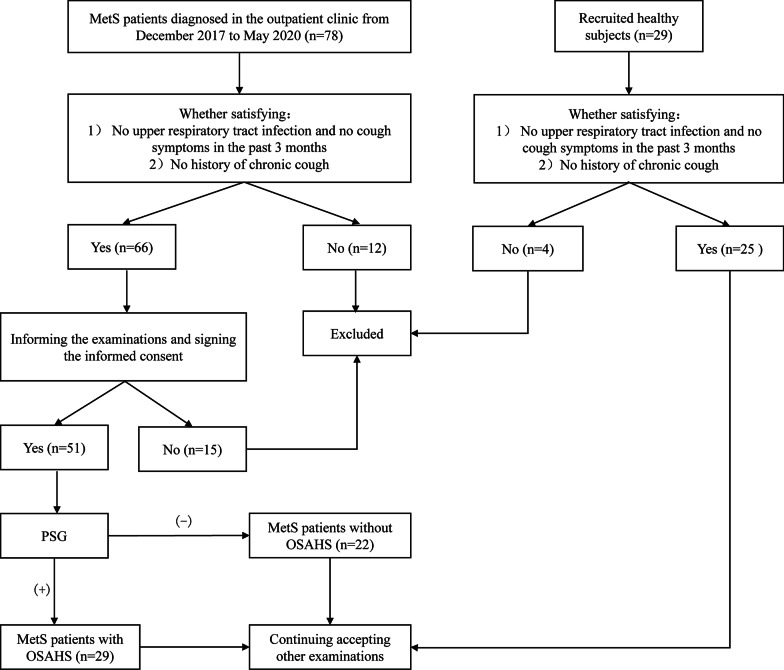
The inclusion process of the three groups of participants of MetS with OSAHS, MetS without OSAHS and healthy control group

The enrollment process was as shown in Fig. 1. The enrolled participants first completed a basic medical history inquiry, had their body height and blood pressure recorded and underwent blood biochemical tests to establish a preliminary diagnosis of MetS; these procedures were followed by PSG monitoring, a pulmonary function test, an exhaled nitric oxide test, a capsaicin cough sensitivity test, and an induced sputum cell count. The remaining sputum was used for cytological analysis and an assay of the supernatant to determine the inflammatory mediator profile. Participants were subsequently divided into a MetS with OSAHS group and a MetS without OSAHS group based on PSG monitoring. The above tests were also completed in the healthy controls.

### Statistical analysis

Data with a normal distribution are expressed as the mean ± SD, while those with a skewed distribution are expressed as the median (25–75% interquartile). Data among the 3 groups were analyzed using one-way analysis of variance (ANOVA) or a nonparametric test (Kruskal–Wallis H test). If there were differences among the groups, the LSD t-test or Mann–Whitney U test was used for pairwise comparisons between 2 groups and categorical variables were compared using the chi-square test. Once differences were determined, we incorporated the most significant individual variables into a stepwise multiple linear regression to determine significant predictors of increasing capsaicin cough sensitivity. (A multiple linear regression model was used to analyze the effects of triglycerides, age, gender, fasting glucose, GerdQ score, FENO, AHI, systolic blood pressure, pepsin concentration and BMI on cough sensitivity.) Correlation analyses were performed using Spearman's rank correlation coefficient. Prospective statistical power calculation based on our published observation [9, 20, 21] indicated that the minimum of 20 patients per group would be required to provide 80% power between two groups using a 5% two‐sided test. Statistical analysis was performed with SPSS version 20.0 (a relatively newer version). *P* < 0.05 was considered statistically significant.

## Results

### General information, pulmonary function indicators and PSG monitoring results for the 3 groups

The general information, pulmonary function indicators, C5 and PSG monitoring results for each group are provided in Table 1. Waist circumference, systolic pressure, diastolic pressure, triglycerides, high-density lipoprotein-cholesterol, and fasting blood glucose were significantly different among the 3 groups. The AHI, minimum oxygen saturation, and average oxygen saturation were significantly different among the 3 groups. The BMI for the 3 groups was significantly different (F = 13.892, *P* = 0.000). The BMI for the MetS with OSAHS group was significantly higher than that for the MetS without OSAHS and the control groups (*P* = 0.001, *P* = 0.000). The GerdQ scores were significantly different among the 3 groups (F = 11.604, *P* = 0.000) and were significantly higher in the MetS with OSAHS and the MetS without OSAHS groups than in the control group (*P* = 0.000, *P* = 0.007). ESS scores were significantly different among the 3 groups (H = 19.985, *P* = 0.000). The ESS score for the MetS with OSAHS group was higher than those for the MetS without OSAHS and control groups (*P* = 0.000, *P* = 0.000).

**Table 1:** Comparison of the general data, pulmonary function indicators, C5, and PSG results among the groups

Variables	MetS with OSAHS	MetS without OSAHS	Healthy subjects
Case number (male)	29 (18)	22 (15)	25 (13)
Age	42.3 ± 10.9	43.3 ± 9.5	38.0 ± 11.8
BMI (kg/m^2^)	27.2 ± 2.8^#▲^	24.3 ± 2.1	22.8 ± 1.9
Neck circumference (cm)	41.6 ± 2.6	38.5 ± 3.4	38.6 ± 3.6
Waist circumference (cm)			
Male	110.1 ± 12.1^#^	108.1 ± 10.1^#^	80.3 ± 2.8
Female	101.8 ± 8.3^#^	97.0 ± 7.2^#^	70.1 ± 9.8
SBP (mmHg)	152.9 ± 10.8^#^	151.8 ± 10.5^#^	124.2 ± 2.5
DBP (mmHg)	90.5 ± 6.4^#^	91.9 ± 6.2^#^	73.2 ± 8.3
Triglyceride (mmol/L)	4.01 ± 1.51^#^	3.42 ± 0.92^#^	1.38 ± 0.23
HDL-C (mmol/L)	0.99 ± 0.03^#^	1.01 ± 0.04^#^	1.95 ± 0.05
Fasting glucose (mmol/L)	7.5 ± 1.6^#^	8.2 ± 1.7^#^	5.3 ± 0.5
GerdQ score (points)	9.1 ± 3.3^#^	8.7 ± 3.5^#^	6.1 ± 0.2
ESS score (points)	9.0 (4.0)^#▲^	3.5 (2.0)	2 (2.0)
Cough symptom score			
Daytime (points)	0 (0)	0 (0)	0 (0)
Night-time (points)	0 (0)	0 (0)	0 (0)
FVC/predicted value%	90.2 ± 9.7	91.2 ± 10.4	92.2 ± 11.3
FEV1/predicted value%	93.1 ± 11.1	94.3 ± 12.7	94.5 ± 8.6
FEV1/FVC%	82.7 ± 9.2	84.1 ± 9.9	83.9 ± 8.6
FeNO (ppb)	17.0 (14.5)	16.0 (11.0)	14.0 (8.5)
C5(μmol/L)	7.80 (13.65)^#^	7.80 (11.70)^#^	15.60 (15.60)
AHI (times/h)	36.5 ± 10.2^#▲^	6.2 ± 2.2	3.7 ± 1.6
Minimum oxygen saturation (%)	65.7 ± 11.3^#▲^	84.1 ± 5.3	89.6 ± 3.2
Average oxygen saturation (%)	81.2 ± 11.7^#▲^	91.5 ± 10.3	92.4 ± 8.9
Nephritis history (n, %)	5 (17.2)	3 (13.6)	2 (8.0)
History of gastric disease (n, %)	8 (27.6)	5 (18.2)	3 (12.0)

*MetS* metabolic syndrome, *OSAHS* obstructive sleep apnea–hypopnea syndrome, *BMI* body mass index, *SBP* systolic blood pressure, *DBP* diastolic blood pressure, *HDL-C* high-density lipoprotein cholesterol, *GerdQ* gastroesophageal reflux disease questionnaire, *ESS* Epworth sleepiness scale, *FVC* forced vital capcacity, *FEV1* forced expiratory volume in one second, *FeNO* fractional exhaled nitric oxide, *C5* the minimum concentrations of inhaled capsaicin that induced ≥ 5 coughs, *AHI*: apnea–hypopnea index

^▲^Compared with the MetS without OSAHS group *P* < 0.05

^#^Compared with the healthy control group *P* < 0.05

### Comparison of capsaicin cough sensitivity among the groups

There was a significant difference in C5 among the 3 groups (H = 14.393, *P* = 0.001). The C5 concentrations for the MetS with OSAHS and MetS without OSAHS groups were significantly lower than that for the control group (*P* = 0.001, *P* = 0.001); however, there was no difference between the 2 MetS groups (*P* = 0.750).

### Comparison of induced sputum cell counts and cell classification among the groups

**Fig. 2:**
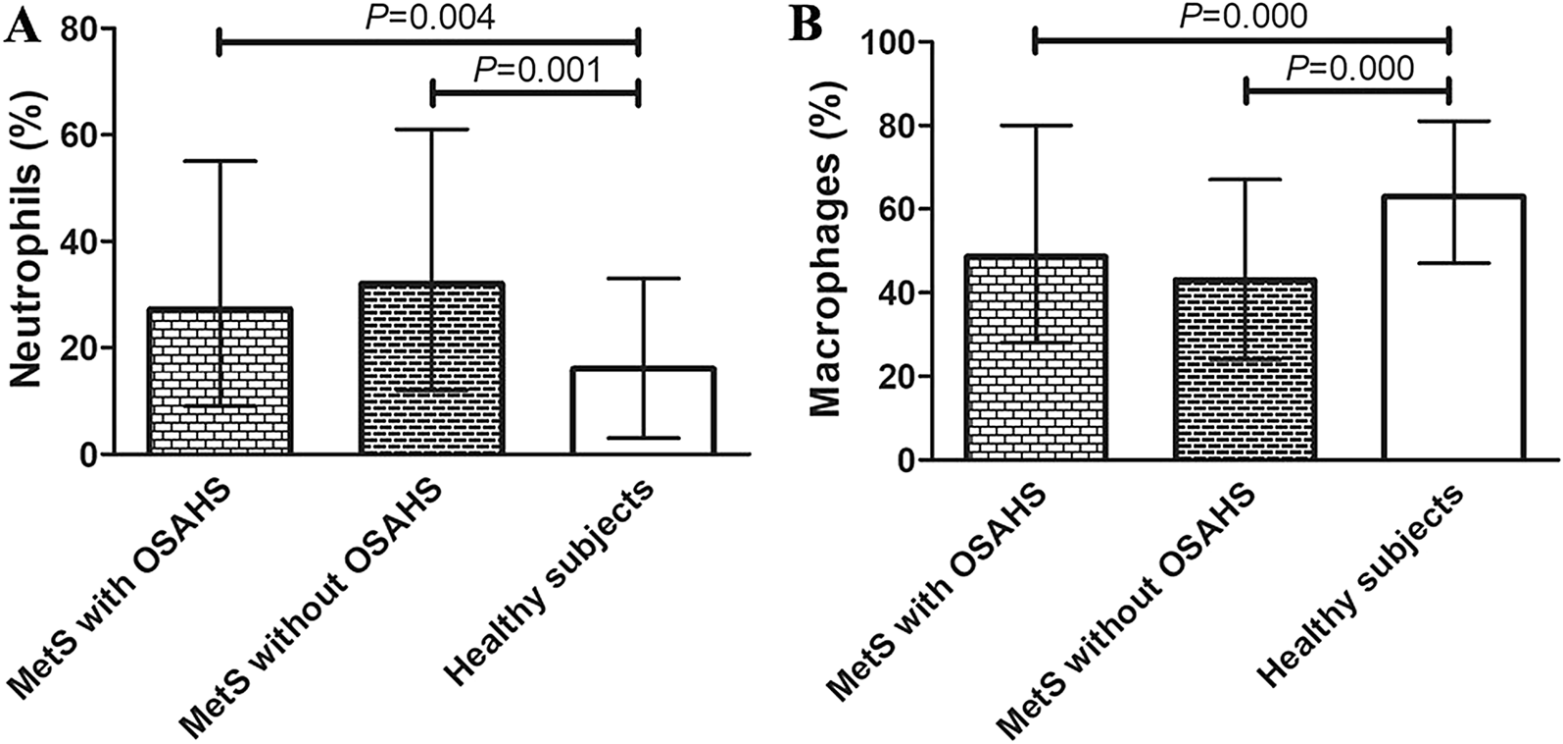
The proportion of neutrophils (**a**) and macrophages (**b**) in induced sputum

There was a significant difference in the proportion of neutrophils among the 3 groups (H = 14.056, *P* = 0.001). The proportion of neutrophils in the MetS with OSAHS and MetS without OSAHS groups was significantly higher than that in the control group (*P* = 0.004, *P* = 0.001); however, there was no difference between the 2 MetS groups (*P* = 0.168, Fig. 2a). The proportion of macrophages were significantly different among the 3 groups (H = 21.468, *P* = 0.000) and significantly lower in the MetS with OSAHS and MetS without OSAHS groups than in the control group (healthy subjects) (*P* = 0.000, *P* = 0.000); however, there was no difference between the 2 MetS groups (*P* = 0.372, Fig. 2b).

### Comparison of inflammatory mediators in the supernatant of induced sputum among the groups

There was a significant difference in CGRP concentration among the 3 groups (F = 17.697, *P* = 0.000). The CGRP concentration in the MetS with OSAHS and MetS without OSAHS groups was significantly higher than that in the control group (*P* = 0.000, *P* = 0.000). The SP concentration was significantly different among the 3 groups (F = 5.892, *P* = 0.008) and significantly higher in the MetS with OSAHS and MetS without OSAHS group than that in the control group (*P* = 0.002, *P* = 0.038). There was a significant difference in IL-8 concentration among the 3 groups (F = 14.340, *P* = 0.000). IL-8 concentration was significantly higher in the MetS with OSAHS and MetS without OSAHS groups than that in the control group (*P* = 0.000, *P* = 0.000). There was a significant difference in pepsin concentration among the 3 groups (F = 129.362, *P* = 0.000). The pepsin concentration in the MetS with OSAHS group was significantly higher than other two groups (Table 2).

**Table 2:** Comparison of inflammatory mediators in the supernatant of induced sputum among the groups

	MetS with OSAHS	MetS without OSAHS	Healthy subjects
Bradykinin (pg/mL)	153.52 ± 27.81	149.22 ± 23.95	133.63 ± 19.28
CGRP(pg/mL)	33.67 ± 2.51^#^	36.50 ± 2.29^#^	19.75 ± 2.12
SP(pg/mL)	42.30 ± 5.12^#^	38.18 ± 6.21^#^	31.33 ± 5.26
Pepsin(ng/mL)	4.36 ± 0.85^#▲^	3.40 ± 0.69^#^	2.18 ± 0.43
PGE_2_(pg/mL)	24.72 ± 5.32	22.85 ± 4.11	18.93 ± 4.83
Histamine (ng/mL)	3.02 ± 0.45	2.86 ± 0.39	2.67 ± 0.26
ECP(ng/mL)	5.51 ± 1.01	5.93 ± 1.12	5.08 ± 0.94
IL-8(pg/mL)	43.50 ± 7.22^#^	45.65 ± 6.87^#^	24.63 ± 3.66

*CGRP* calcitonin gene-related peptide, *SP* substance P, *PGE2* prostaglandin E2, *ECP* eosinophil cationic protein, *IL-8* interleukin-8

^▲^Compared with the MetS without OSAHS group *P* < 0.05

^#^Compared with the healthy control group *P* < 0.05

### Correlation analysis of capsaicin cough sensitivity and each indicator in the MetS population

**Fig. 3:**
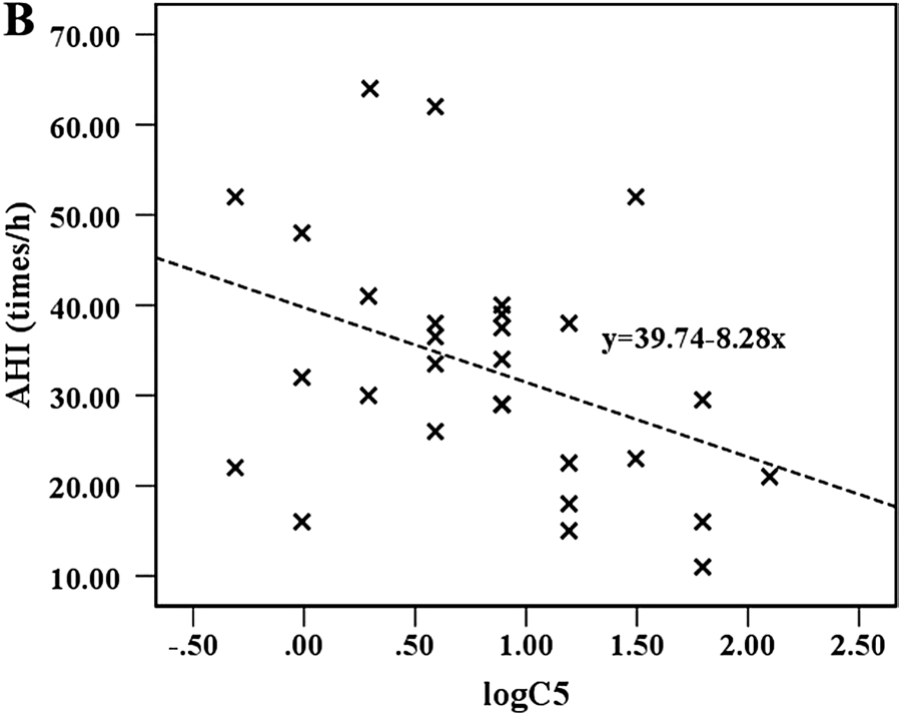
Correlation between lgC5 and the AHI in the MetS with OSAHS group

The correlation between capsaicin cough sensitivity (lgC5) and various indicators in the MetS with OSAHS and MetS without OSAHS groups were analyzed. Spearman rank correlation analysis showed that in the MetS with OSAHS group, lgC5 was negatively correlated with the AHI (r = − 0.394, *P* = 0.035, Fig. 3) and was not correlated with triglycerides, HDL-C, fasting blood glucose, bradykinin, CGRP, SP, pepsin, IL-8 and other indicators. In the MetS without OSAHS group, there was no correlation between lgC5 and the aforementioned indicators. In terms of exogenous factors, there was no correlation between lgC5 and waist circumference, blood pressure, GerdQ score, ESS score, neck circumference, history of past rhinitis, history of past gastritis, FeNO, lung function indicators, regardless of whether MetS patients had OSAHS or not.

### The multiple linear regression of capsaicin cough sensitivity

Triglycerides, age, gender, fasting glucose, GerdQ score, FENO, AHI, systolic blood pressure, pepsin concentration and BMI were included as independent variables into the multiple linear regression. The results showed that triglycerides, AHI, pepsin concentration and BMI were negatively correlated with cough threshold which as shown in Table 3.

**Table 3:** The multiple linear regression of capsaicin cough sensitivity

	B	Beta	t	*P*
Constant	9.533		11.937	0.000
Triglyceride	− 0.332	− 0.251	− 2.245	0.028
Age	0.002	0.012	0.161	0.873
Gender	− 0.375	− 0.091	− 1.217	0.228
Fasting glucose	0.032	0.028	0.327	0.744
GerdQ score	0.022	0.032	0.395	0.694
FENO	− 0.083	− 0.021	− 0.289	0.773
AHI	− 0.026	− 0.207	− 2.384	0.020
SBP	− 0.167	− 0.090	− 0.961	0.340
Pepsin	− 0.472	− 0.250	− 2.433	0.018
BMI	− 0.594	− 0.282	− 3.282	0.002

*BMI* body mass index, *SBP* systolic blood pressure, *GerdQ* gastroesophageal reflux disease questionnaire, *FeNO* fractional exhaled nitric oxide, *AHI* apnea–hypopnea index

## Discussion

This study found that the capsaicin cough sensitivity was increased in MetS patients independent of the presence OSAHS, manifesting as a decreased cough threshold accompanied by an increased percentage of neutrophils in induced sputum and an increased concentration of IL-8, SP, CGRP and pepsin in the supernatant; additionally, the GerdQ scores for MetS patients was significantly higher than those for the control group. The caspase cough thresholds lgC5 for the MetS with OSAHS group were significantly negatively correlated with the AHI. Triglyceride, AHI, pepsin concentration and BMI are risk factors for increased capsaicin cough sensitivity and the pepsin concentration in the MetS with OSAHS group was higher than that in the MetS without OSAHS group.

MetS is the general term for the presence of multiple clinical diseases, including obesity, hypertension, insulin resistance (or type 2 diabetes) and dyslipidemia. Data from China indicate that the prevalence of MetS among people over 60 years of age is 58.1% [22]. With an increase in BMI, the prevalence of OSAHS also significantly increases; therefore, MetS is closely related to OSAHS [23]. A study found that 33% of OSAHS patients had chronic cough, of which 28% had nighttime heartburn symptoms and 44% had rhinitis symptoms, suggesting that OSAHS-associated gastroesophageal reflux and postnasal drip may cause long-term cough [24]. Our previous studies have also suggested that OSAHS patients have a high sensitivity to cough, manifesting as cough susceptibility [9]. This study indicated that the capsaicin cough sensitivity of the MetS with OSAHS group was higher than that of the control group. The capsaicin cough sensitivity of the MetS with OSAHS group was negatively correlated with the AHI, suggesting that increased capsaicin cough sensitivity in MetS patients is closely related to OSAHS. The mechanism may be caused by airway inflammation, especially airway neurogenic inflammation and repeated airway obstruction [9]. Gastroesophageal reflux is a common cause of chronic cough [25, 26], and OSAHS patients have increased negative intrathoracic pressure during apnea, which induces or aggravates gastroesophageal reflux. Studies have found that nighttime reflux events in OSAHS patients are increased and that the incidence of nighttime reflux events is positively correlated with the AHI [27]. In this study, the pepsin concentration in the MetS with OSAHS group was higher than that in the MetS without OSAHS group, indicating that in MetS patients, OSAHS may aggravate the severity of gastroesophageal reflux. The results of this study also indicated that history of rhinitis, concentrations of ECP, and histamine in induced sputum were not significantly different among the 3 groups, suggesting that the increase in capsaicin cough sensitivity in MetS patients was not significantly related to rhinitis.

Our study also found that the capsaicin cough sensitivity of patients with MetS without OSAHS significantly increased, indicating that increased cough sensitivity in MetS patients was not simply caused by the presence of OSAHS. The pepsin concentration and the GerdQ score in the MetS without OSAHS group were significantly higher than those in the control group, suggesting that gastrointestinal reflux is an important factor for the increase in capsaicin cough sensitivity in MetS patients without OSAHS. Why did these patients also have gastroesophageal reflux? First, hypertriglyceridemia is a risk factor for gastroesophageal reflux [28–31]. The probable reason is that the lower esophageal sphincter pressure and the autonomic contraction frequency in the lower esophagus are affected by elevated blood lipid levels, leading to the abnormal secretion of gastrointestinal hormones and an increase in low-density lipoproteins, subsequently inducing gastroesophageal reflux. Second, high blood glucose levels can cause autonomic neuropathy, which in turn affects esophageal motility and gastrointestinal emptying. In patients with diabetes, esophageal motility is reduced, peristaltic waves are reduced or absent, reverse peristalsis can occur, and gastroesophageal reflux-related symptoms are present [32, 33]. In addition, hypertension and gastroesophageal reflux have many common risk factors, such as age, obesity, smoking, and drinking [31, 34, 35]. However, the specific mechanism is still not clear. The mechanism of chronic cough caused by gastroesophageal reflux includes the theory of reflux and the theory of reflex. The former proposes that reflux of the stomach contents into the gullet or trace amounts of aspiration into the lungs can stimulate the throat or trachea and tracheobronchial cough receptor to cause coughing. The latter proposes that esophageal stimuli generated by lower esophageal reflux induce airway neurogenic inflammation through an esophageal-bronchial reflex and stimulate coughing centers or a sensitize cough reflex to cause coughing. We further explored the factors affecting the cough sensitivity of MetS patients through a multiple linear regression model, and finally confirmed triglyceride, AHI, pepsin concentration and BMI as the risk factors, which verified our conjecture to a certain extent.

This study found that neutrophilic inflammation exists in the lower airway of MetS patients. Taking into account the steps for cleaning the nasal cavity and mouth during the induction process, the possibility of contamination of nasopharyngeal inflammatory secretions in sputum is relatively low. Therefore, the neutrophils in the induced sputum are mainly from the lower airway. The patients in the MetS group did not have infection-related manifestations, such as fever and jaundice. Therefore, neutrophilic airway inflammation was not caused by infection. Coughing itself can cause mechanical injuries to the airway mucosa, and related studies in a guinea pig cough model, including those by our group, also showed that coughing can cause airway damage, produce neutrophilic airway inflammation, and increase the hypersensitivity of the cough reflex [36, 37]. Activated neutrophils can secrete neutrophil elastase (NE) to further promote the release of IL-8 from epithelial cells. In this study, the increase in IL-8 concentration in the supernatant of induced sputum from MetS patients also provided evidence of neutrophilic airway inflammation. In addition, neutrophil metabolism is related to the expression of receptor for advanced glycation end products (RAGE) [38, 39]. RAGE is a cell membrane surface protein. After binding with the corresponding ligands, RAGE upregulates the synthesis and secretion of IL-8 and promotes the local infiltration of neutrophils. Furthermore, it inhibits the phagocytosis of apoptotic neutrophils by phagocytic cells, directly leading to the release of antigenic substances in apoptotic cells, thereby aggravating the local inflammatory response [40, 41]. Further studies need to be conducted to confirm the upregulation of RAGE expression in the lower airway of MetS patients, which results in an increase in the proportion of neutrophils. The classical mechanism of cough hypersensitivity involves the inflammatory-mediator activation of TRPV1, which stimulates the vagal afferent terminals, resulting in neurogenic airway inflammation, leading to the release of neuropeptides such as SP and CGRP which stimulates local nerves as well as transmitting signals to the central nervous system, thereby increasing cough sensitivity [42]. The results of this study showed that the concentrations of SP and CGRP in the supernatant of induced sputum from MetS patients significantly increased, further indicating that airway inflammation, especially neurogenic airway inflammation, plays an important role in the cough hypersensitivity of MetS patients.

This study has some limitations. First, induced sputum from MetS patients was not retested after treatment to further confirm the causal relationship between neutrophilic airway inflammation and cough. Second, all participants did not receive multichannel intraluminal impedance combined with pH monitoring (MII). The specific reflux property and severity were not clear, but we explored the pepsin concentration in the induced sputum supernatant and the GerdQ score and evaluated gastroesophageal reflux from the aspects of objective detection and subjective evaluation. Third, related inspections for cough susceptibility, including cough induced by inhalation of capsaicin, induced sputum testing, lung function, FeNO, PSG monitoring, are not routine examination items for patients with metabolic syndrome, leading to difficulties in inclusion and insufficient sample size. We will continue to expand the sample size and conduct further research.

In summary, MetS patients are susceptible to cough. Increased capsaicin cough sensitivity in these patients is closely related to sleep apnea and gastroesophageal reflux. In MetS patients without OSAHS, gastroesophageal reflux is an important factor that increases capsaicin cough sensitivity. Airway inflammation, especially airway neurogenic inflammation, may plays a role in increased capsaicin cough sensitivity in MetS patients.

## Data Availability

The datasets used and/or analysed during the current study are available from the corresponding author on reasonable request.
